# Supplementary material for: Inferring the extinction risk of marine fish to inform global conservation priorities
Source: PLoS Biol. 2024 Aug 29;22(8):e3002773. doi: 10.1371/journal.pbio.3002773 (PMC11361419; doi:10.1371/journal.pbio.3002773)

**Supplementary materials of** **Inferring the extinction risk of marine fish to inform global conservation priorities**

Table A: Table summarizing ecological and human-uses traits utilized to predict IUCN status.

| **Traits** | **Type** | **Unit** | **Range or Number of categories** |
| --- | --- | --- | --- |
| growth rate | continuous | NA | 0.008 - 1.14 |
| maximum length | continuous | cm | 2 - 801 cm |
| mode of reproduction | categorical | NA | 4 categories |
| maximum depth | continuous | meters | 1 - 8,371 m |
| minimum depth | continuous | meters | 1 - 4,376 m |
| reproduction fertilization | categorical | NA | 4 categories |
| body shape | categorical | NA | 5 categories |
| trophic level | continuous | NA | 0.47-0.80 |
| climate niche | qualitative | NA | 3 categories |
| price categories | qualitative | NA | 3 categories |
| interest for aquariums | ordered | NA | 4 categories |

Figure A: Boxplot representing the percentages of True predictions (TP), False Positives (FP) and False Negatives (FN) of random forest model (RF) and the artificial neural network algorithm (ANN).


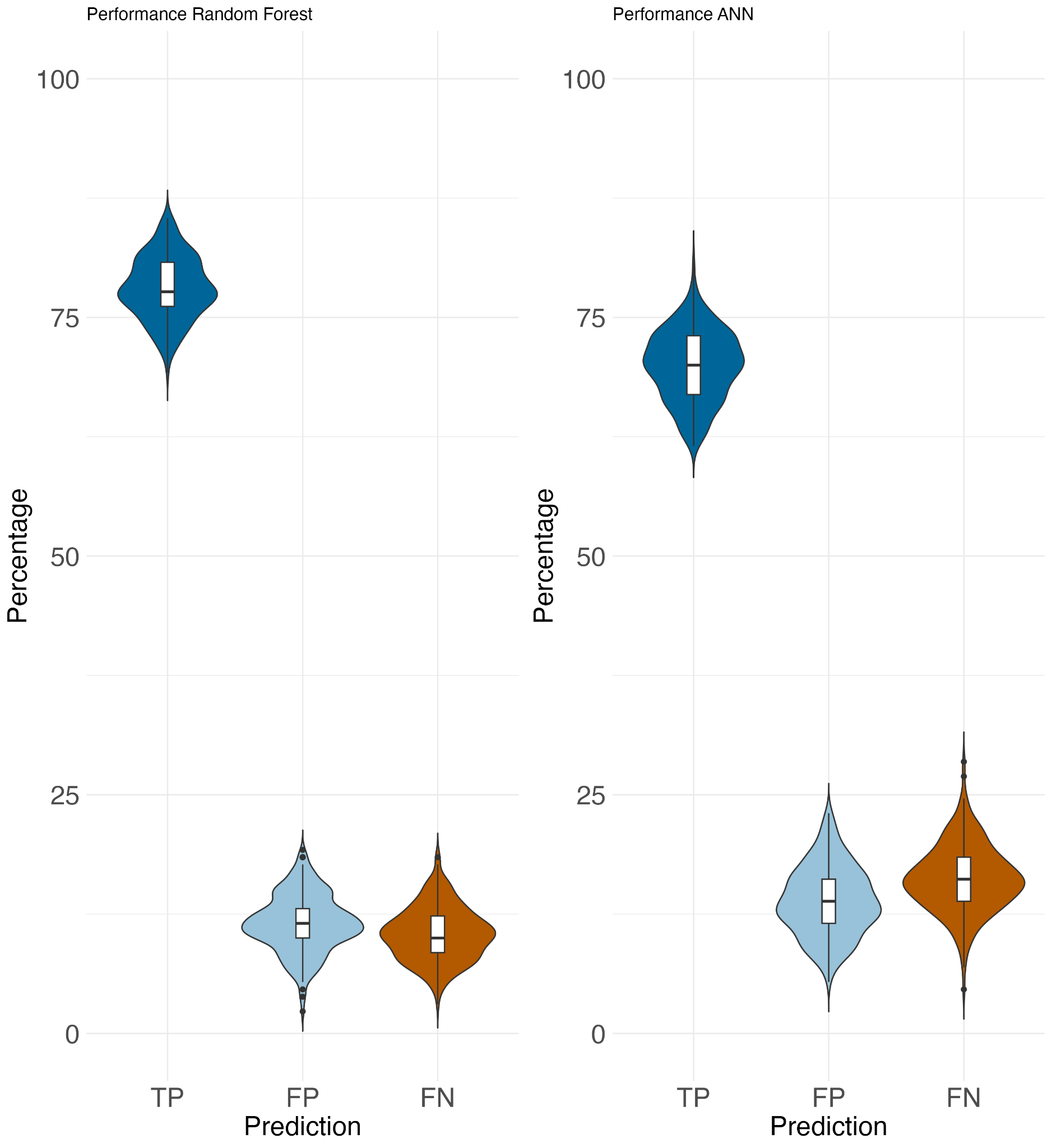


Figure B: Boxplot representing performance statistics - Accuracy, F1, recall, precision scores of random forest model (RF) and the artificial neural network algorithm (ANN).


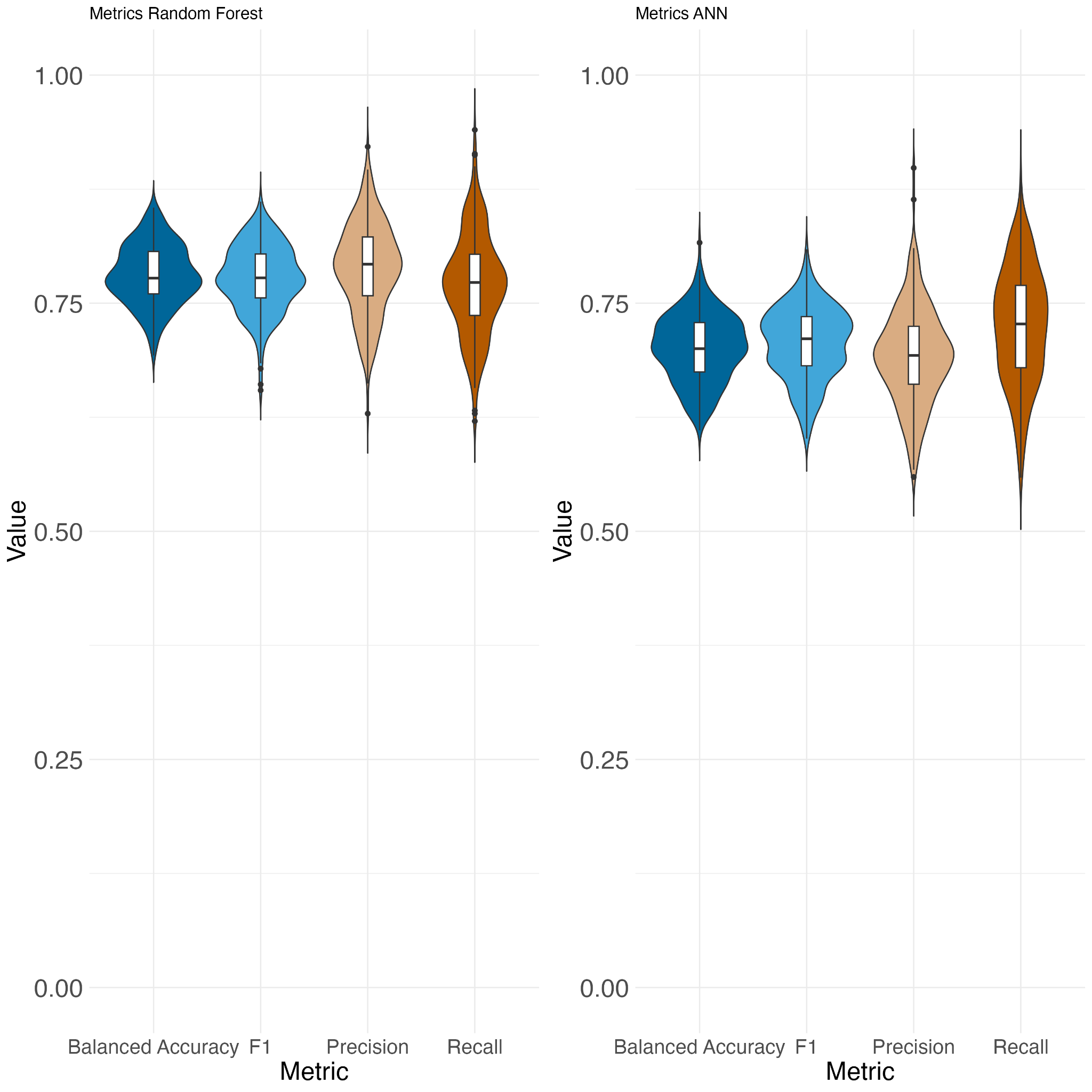


Figure C: Chord diagram showing the distribution of species within the different categories before and after prediction when ANN and random forests outputs are used in a consensus way. Threatened” (red) including Critically Endangered, Endangered, and Vulnerable species; “Non-Threatened” (in blue) including Least Concern and Near Threatened species; “No Status” (DDNE; in yellow) merging Data Deficient and Not Evaluated species. 824 species were predicted as threatened, 1,846 as non-threatened and 2,322 species remained DDNE.


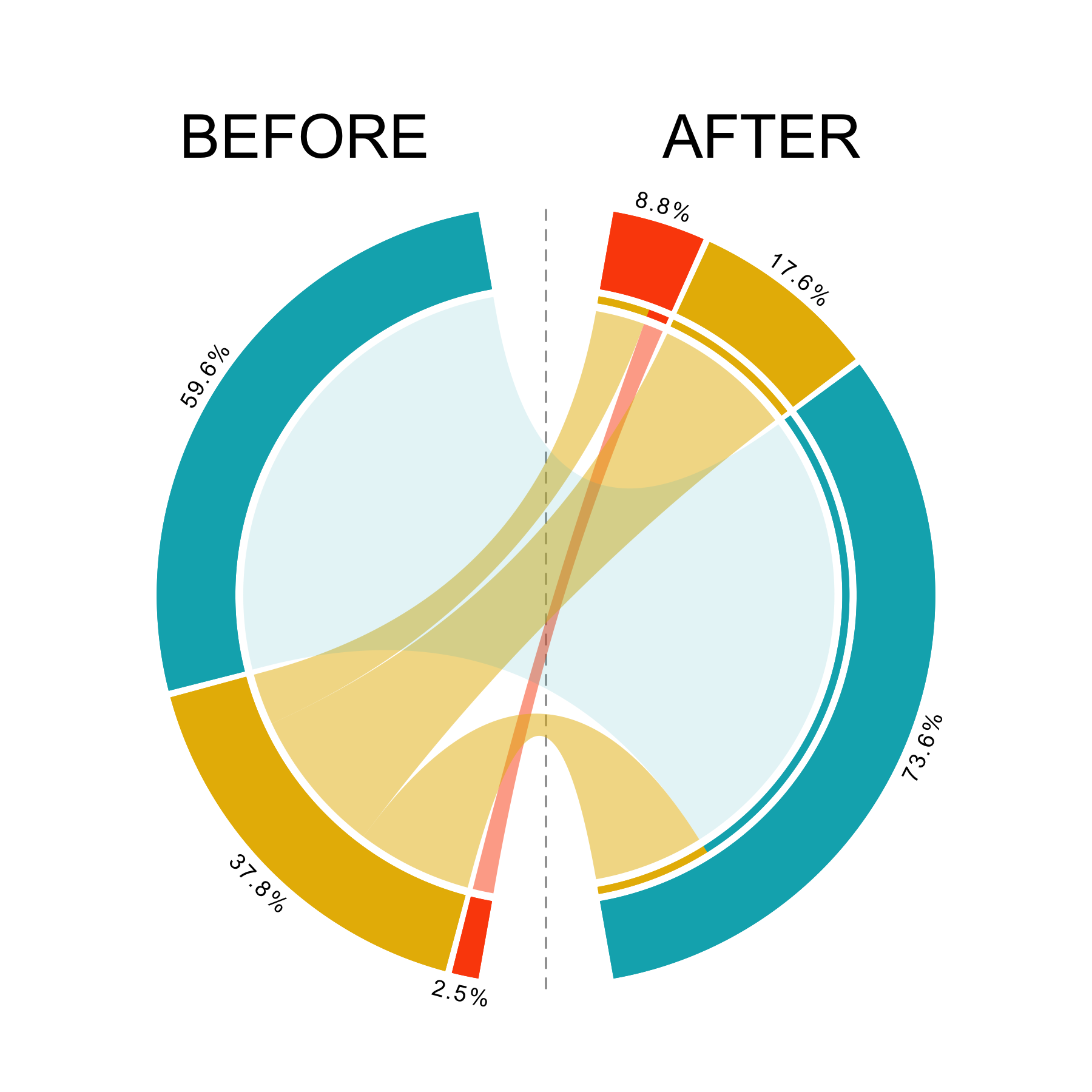


Figure D: IUCN categories of the 4,992 predicted fishes mapped over the phylogeny. Distribution of the values of phylogenetic signals of ecological rarities (index D) computed on 100 trees are plotted in the center of the tree. The figure represents a single phylogeny from the 100 phylogenies generated (see “Methods”). Threatened” (red) including Critically Endangered, Endangered, and Vulnerable species; “Non-Threatened” (in blue) including Least Concern and Near Threatened species; “No Status” (DDNE; in yellow) merging Data Deficient and Not Evaluated species and “Non predicted” species in gray.


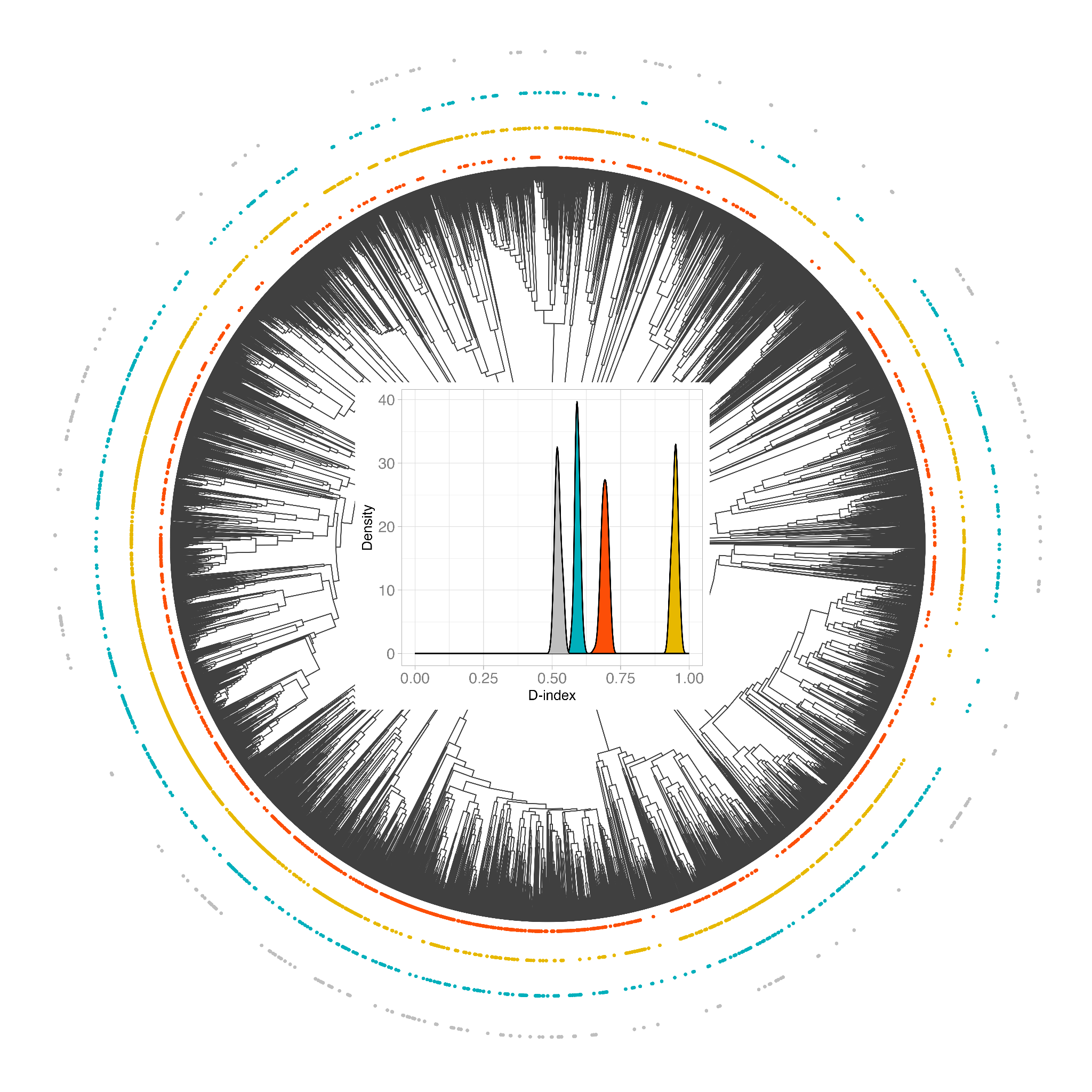


Figure E: Number of species per family predicted as threatened, non-threatened and that remained no-status. Grey represented species with too many trait missing values and not incorporated in the predictive framework. Species are ordered by the number of threatened species.


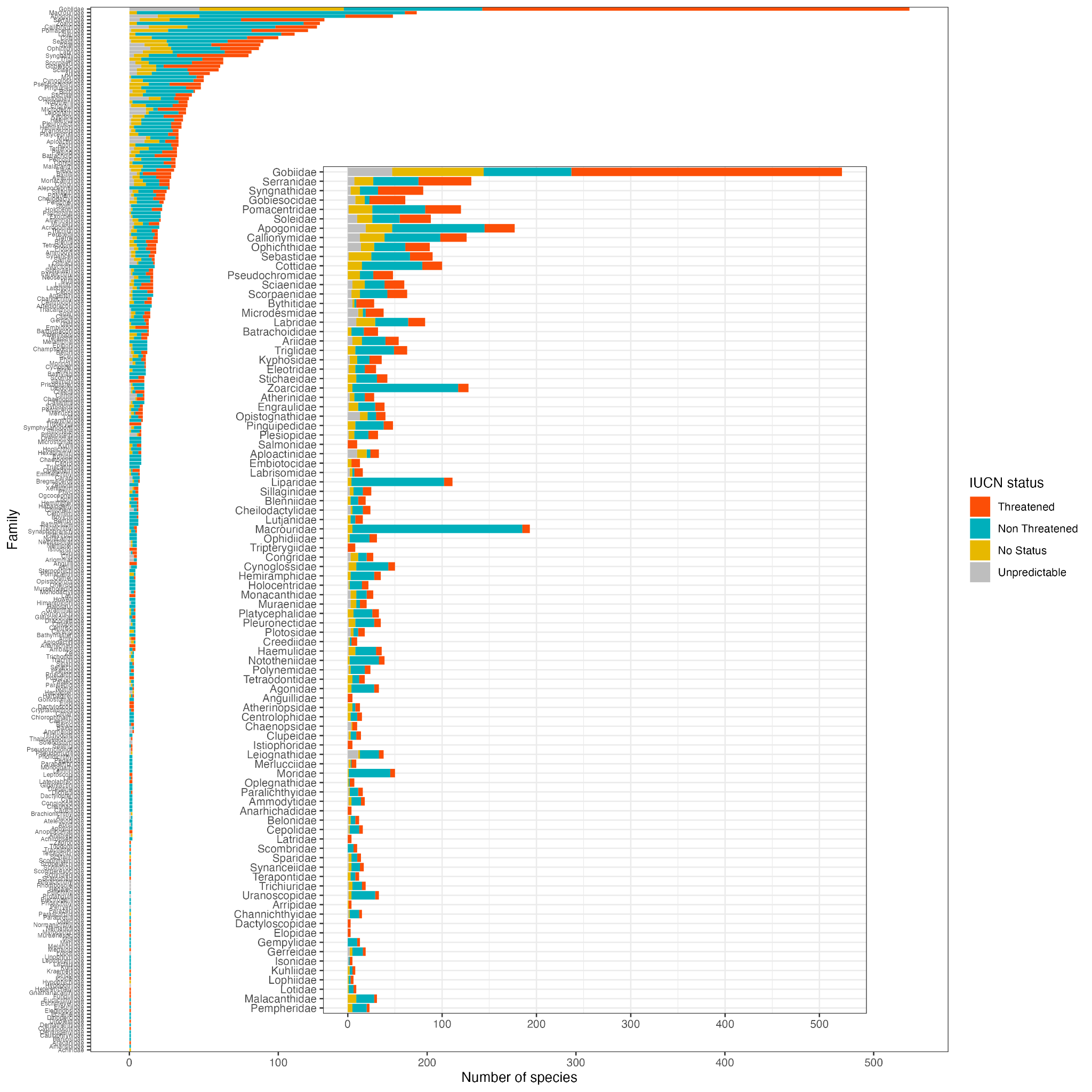


Figure F: Spatial distribution of the difference in number of Threatened species, Non- Threatened species, and DDNE species before and after prediction (consensus decision tree framework).


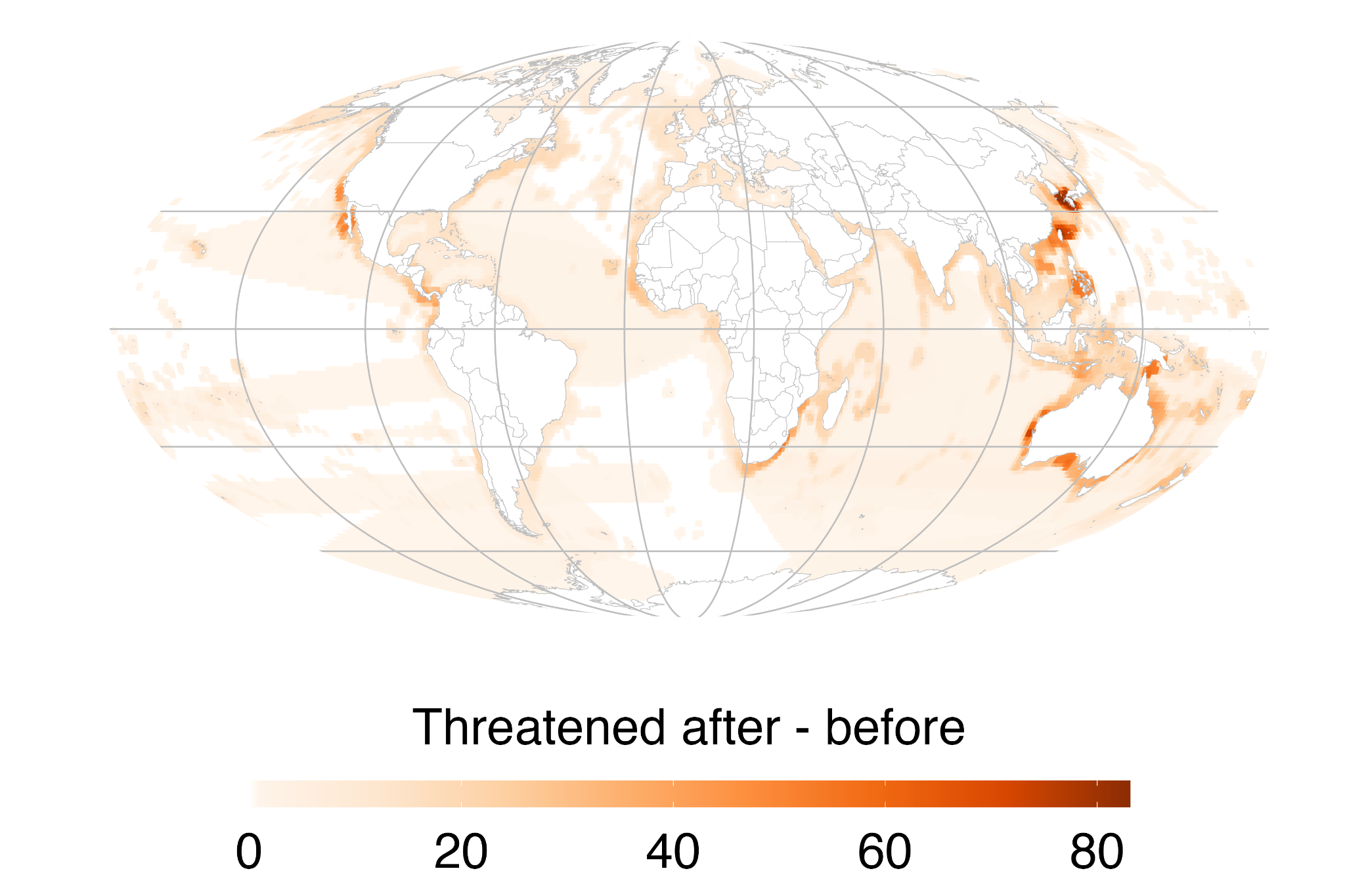


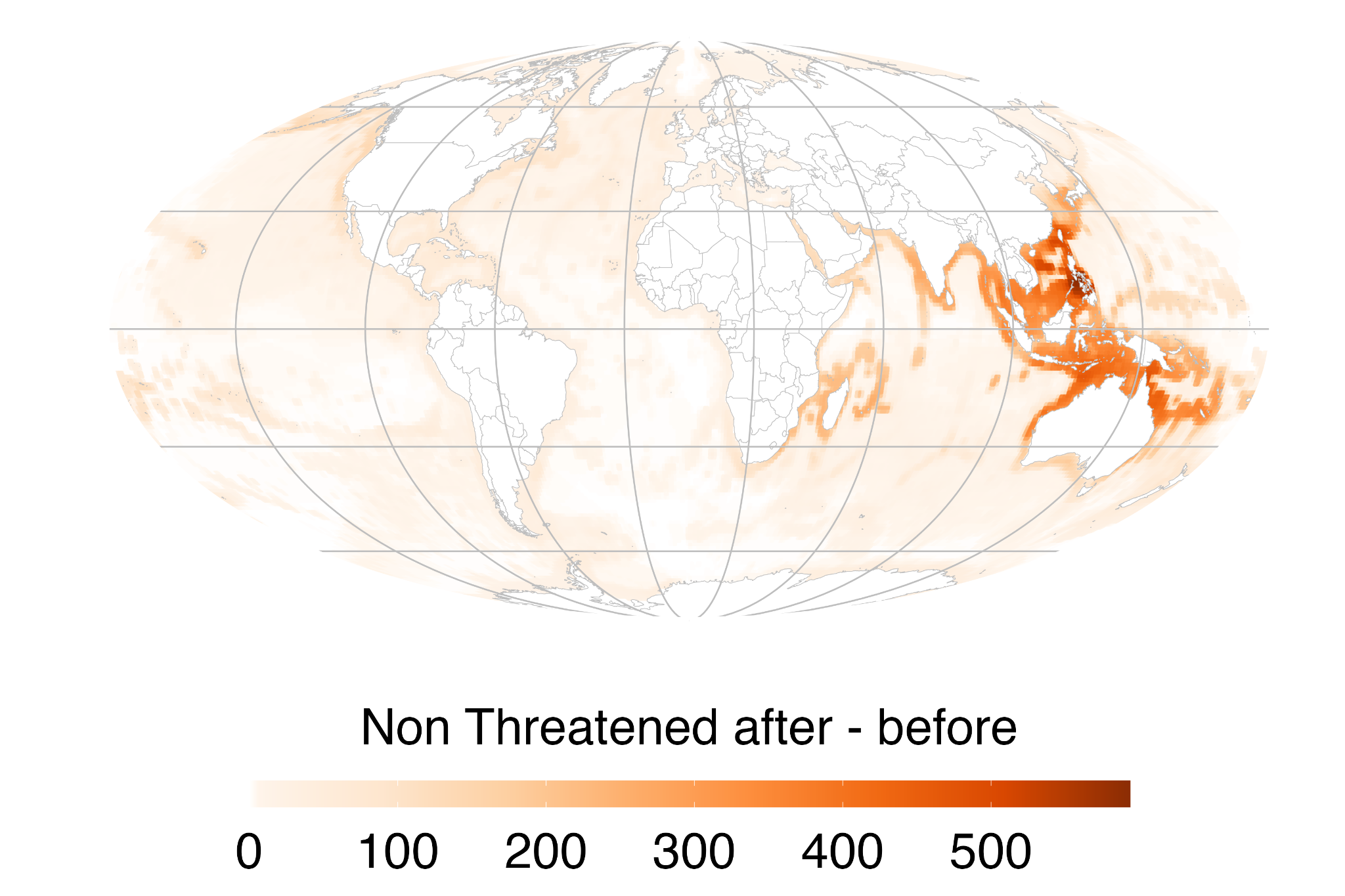


*
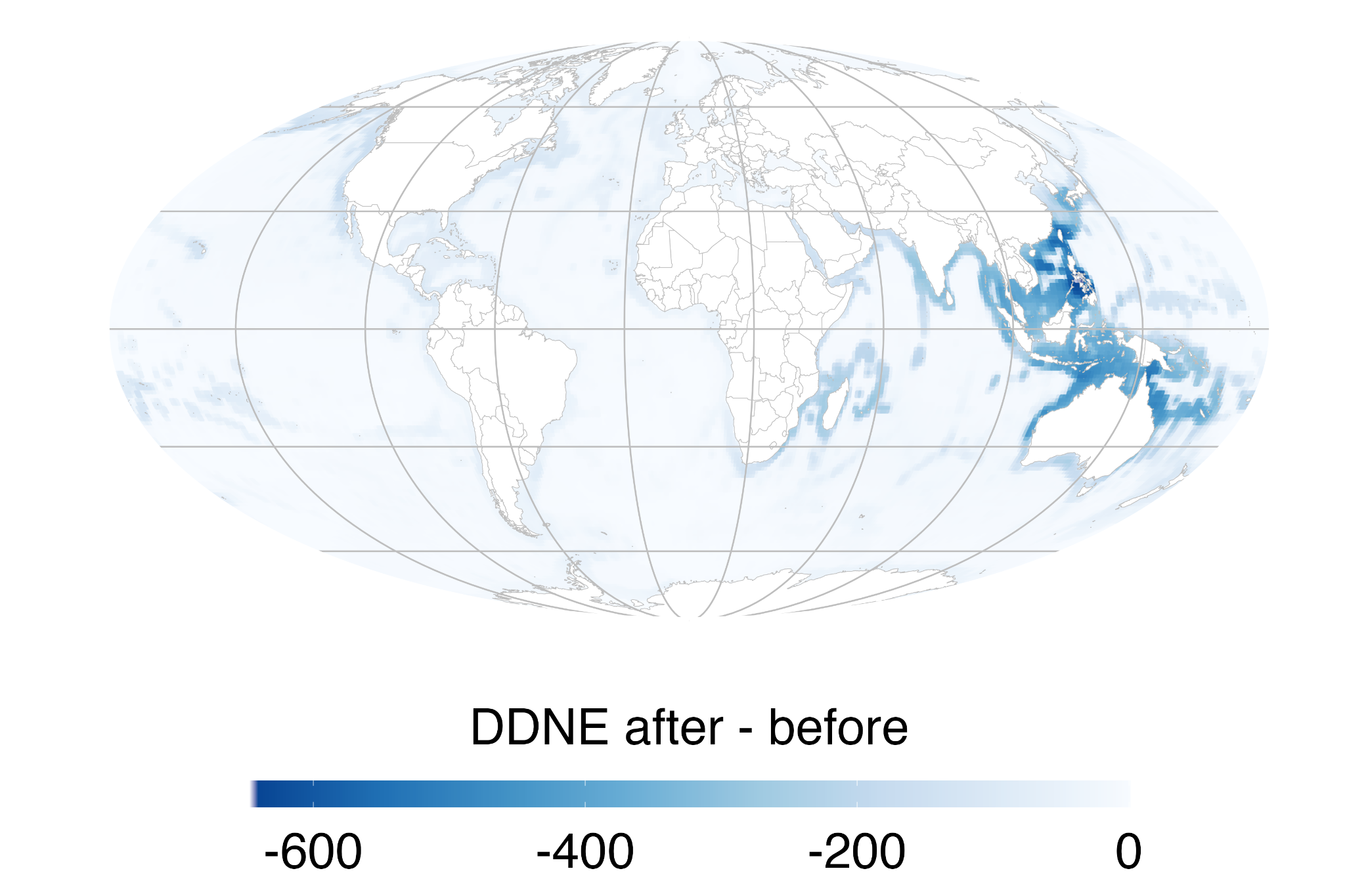
*

Figure G: Robustness analyses of change in Zonation priority results (difference in ranking) for the 13,195 species weighted by their IUCN status before and after prediction. We scored species without any status after prediction as 2. Species predicted as Non-Threatened were scored as 2 minus the percentage of models that predicted the status and species predicted as threatened were scored as 2 plus the percentage of models that predicted the status (scored standardized between 2 and 5). By doing so we weight more species that have been evaluated by IUCN than by our model. Biplot showing the relationship between ranks before and after prediction. Each point represents a cell. Points above x=y mean that priority rank of the given cell increases, while points below x=y mean that priority rank of the given cell decreases after addition of predicted IUCN status, color gradient indicates the species richness of cells.

Secondary plot on the left : relationship between the delta ranks (rank after - rank before) of each cell and species richness (log10). The two red lines show the quantile regression (10% and 90%) using the rqss() (additive quantile regression smoothing) function of the R package quantreg v.5.95; points color gradient indicates the density (log10) of points from high (yellow) to low (blue). Secondary plot on the right: latitudinal gradient of species richness (log10). Points color gradient indicates the density of points from high (yellow) to low (blue).


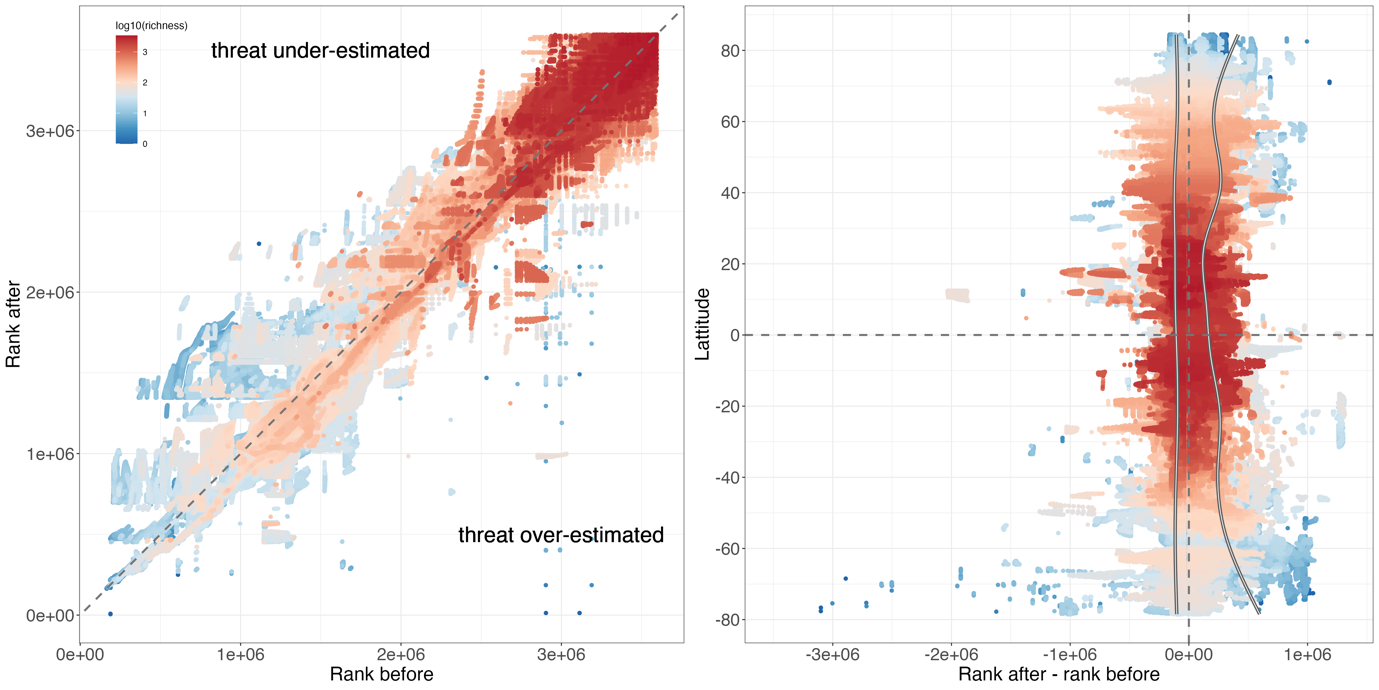


Figure H: **Left** : relationship between the delta ranks (rank after - rank before) of each cell and species richness (log10). The two red lines show the quantile regression (10% and 90%) using the rqss() (additive quantile regression smoothing) function of the R package quantreg v.5.95; points color gradient indicates the density (log10) of points from high (yellow) to low (blue). **Right**: latitudinal gradient of species richness (log10). Points color gradient indicates the density of points from high (yellow) to low (blue).


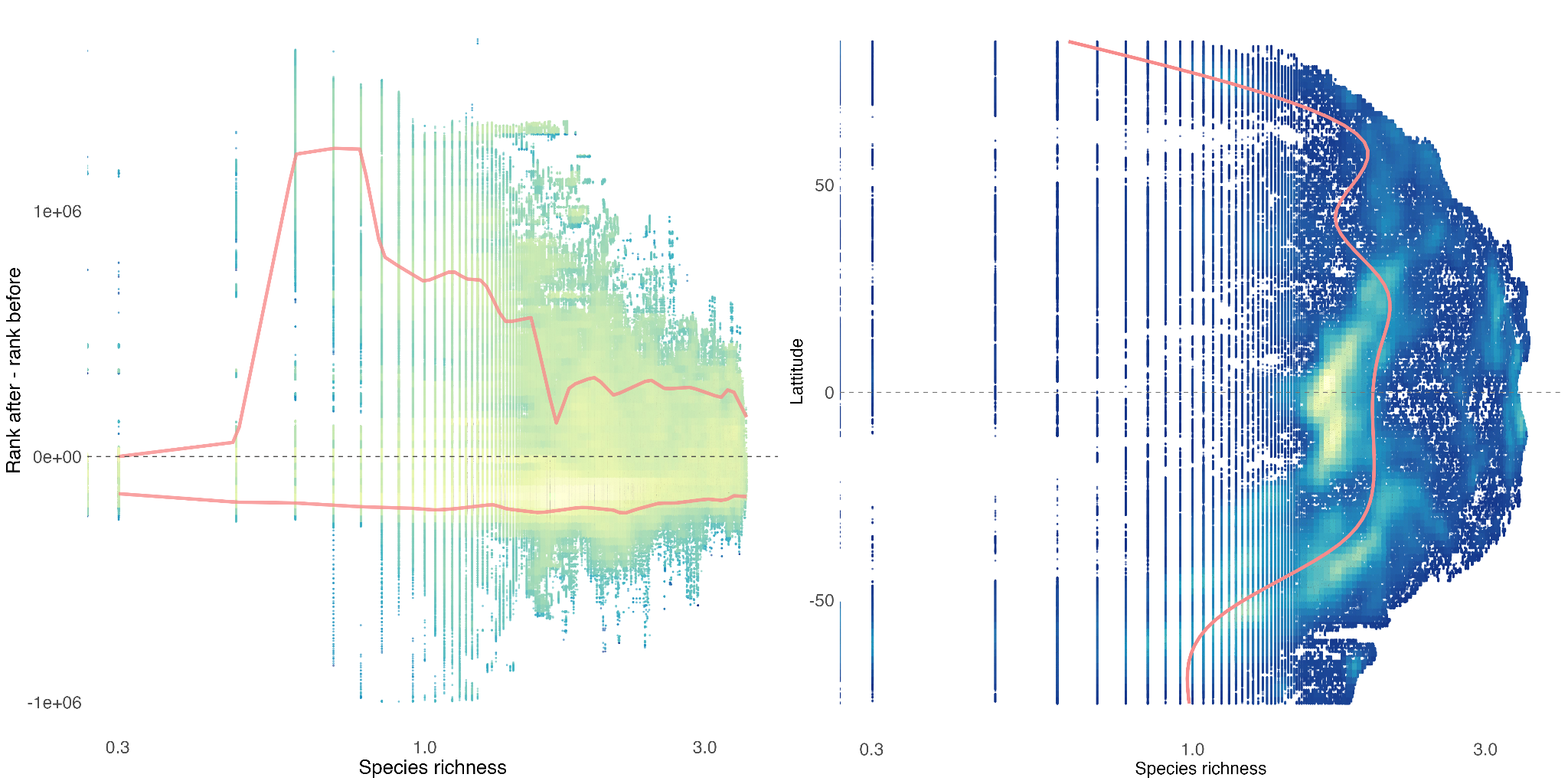

Supplement: S1 Text — Table A in S1 Text. Table summarizing ecological and human-uses traits utilized to predict IUCN status. Figure A in S1 Text. Boxplot representing the percentages of True predictions (TP), False Positives (FP), and False Negatives (FN) of random forest model (RF) and the artificial neural network algorithm (ANN). The data underlying this figure can be found in https://zenodo.org/records/12783687. Figure B in S1 Text. Boxplot representing performance statistics—Accuracy, F1, recall, precision scores of random forest model (RF) and the artificial neural network algorithm (ANN). The data underlying this figure can be found in https://zenodo.org/records/12783687. Figure C in S1 Text. Chord diagram showing the distribution of species within the different categories before and after prediction when ANN and random forests outputs are used in a consensus way. “Threatened” (red) including Critically Endangered, Endangered, and Vulnerable species; “Non-Threatened” (in blue) including Least Concern and Near Threatened species; “No Status” (DDNE; in yellow) merging Data Deficient and Not Evaluated species. A total of 824 species were predicted as threatened, 1,846 as non-threatened, and 2,322 species remained DDNE. The data underlying this figure can be found in https://zenodo.org/records/12783687. Figure D in S1 Text. IUCN categories of the 4,992 predicted fishes mapped over the phylogeny. Distribution of the values of phylogenetic signals of ecological rarities (index D) computed on 100 trees are plotted in the center of the tree. The figure represents a single phylogeny from the 100 phylogenies generated (see “Methods”). “Threatened” (red) including Critically Endangered, Endangered, and Vulnerable species; “Non-Threatened” (in blue) including Least Concern and Near Threatened species; “No Status” (DDNE; in yellow) merging Data Deficient and Not Evaluated species and “Non predicted” species in gray. The data underlying this Figure can be found in https://zenodo.org/records/12 [file pbio.3002773.s002.docx]
