# Supplementary material for: Inferring the extinction risk of marine fish to inform global conservation priorities
Source: PLoS Biol. 2024 Aug 29;22(8):e3002773. doi: 10.1371/journal.pbio.3002773 (PMC11361419; doi:10.1371/journal.pbio.3002773)
Supplement: Attachment — Submitted filename: Response to Reviewers.docx [file pbio.3002773.s003.docx]

Dear Editor,

We are very grateful to the Academic Editor and Reviewers for their positive and constructive comments, and we are pleased to submit the revised version of our manuscript entitled “Inferring marine fish extinction risk to inform global conservation priorities” ( PBIOLOGY-D-23-03393R1)

Here is our answer to the points mentioned by you and the Academic Editor:

*“ You'll see that reviewer #1 is very positive, and only has textual requests, suggesting a movement of material from the Discussion to the Results, wanting comment on the age (10 yrs) of the occurrence data, and asking you to mention climate change more prominently.”*

We chose to work with this global dataset because it is the most accurate, even if not the most recent, on coastal fishes. Indeed, data provided by Albouy et al. (2019) are based on robust methods to minimize errors in the original OBIS data set by removing erroneous occurrences, controlling for depth, and merging with the GASPAR database (Parravicini et al. 2021). Since, by construction, OBIS is continuously aggregating new observations it would be very difficult, if not impossible, to get range contractions. Yet, species with no observation in a given location after 2014 in OBIS are not necessarily absent from this location. For these reasons we believe that our dataset is the most accurate one for all marine fishes available today. We agree that some changes in species distributions over the last 10 years have occurred but we cannot overcome this limitation. We now mention this point in the discussion (lines 321-326).

*Albouy, C. et al. The marine fish food web is globally connected. Nature Ecology Evolution 3, 1153-1161(2019).*

*Parravicini, V., Bender, M. G., Villéger, S., Leprieur, F., Pellissier, L., Donati, F. G. A., ... & Kulbicki, M. (2021). Coral reef fishes reveal strong divergence in the prevalence of traits along the global diversity gradient. Proceedings of the Royal Society B, 288(1961), 20211712.*

“*Reviewer #2 is also positive, but wants several additional (and sensible-sounding) analyses to explore “erroneous” classification, to calculate additional performance stats and to do a sensitivity analysis; they also want clarity around how grid sizes were handled. Reviewer #3 is broadly positive, but raises a number of concerns arising from the combination of algorithms and wants more clarity around sensitivity analyses.*” & “*I discussed these comments with the Academic Editor, who agreed that we should invite a revision to address the concerns raised by the reviewers. The Academic Editor had previously mentioned two concerns;  one is that you should quantify the uncertainty of each assignment (this issue also seems to be raised by reviewer #2)*”

Methodological improvements to evaluate the performance of models as suggested by Reviewers #2 and #3 and Academic Editor have been implemented. Following their relevant comments we calculated additional performance statistics (see supplementary Figure S1 and S2) that enriched our discussion. We also improved the description of complementary and consensus decisions between Random-Forest and Artificial Neural Network algorithms. We added a discussion on the caveats associated with prediction accuracy of our framework (lines 258-303). We made the methodological description of Zonation clearer with a better justification of the weight used both in the method and result sections (lines 246-250,553-568).

*“... you should substantially revise Figure 7: I think it is too busy and log(10)richness should be back-transformed into something more meaningful along with maybe labelling the x-axis to explain "rank after - rank before" more intuitively, e.g. threat under-estimated vs over-estimated at different ends of the x-axis."*

To make figure 7 less busy, we removed the two secondary plots and moved them into supplementary figure S8 We added “threat under-estimated” and “threat over-estimated” on both panels of figure 7 as proposed by the Academic Editor.

Our revised manuscript now uses the Artificial Neural Network (ANN) computed with the R package *cito* rather with Pytorch & Python (as previously) to make our entire pipeline easier to reproduce within a single coding environment (R). This coding change did not change our results.

You’ll find the answers to all comments made by reviewers below (line numbers refer to the revised manuscript without tracked changes, with code RX#Y for reviewer X comment Y). Thank you for your consideration and we look forward to working with you during the editorial process.

Please note that I will be on a field trip from July 1st to the 15th with no internet access. If you have any questions you can email the last author Nicolas Mouquet: [nicolas.mouquet@cnrs.fr](mailto:nicolas.mouquet@cnrs.fr).

All authors have seen and approved the revised version.

Yours sincerely,

Nicolas Loiseau on behalf of all authors

------------------------------------
REVIEWERS' COMMENTS:


***Reviewer #1:*** *[identifies herself as Alice Rogers]

R1#1 This manuscript presents a well-considered and thoroughly explained method to predict and assign extinction risk categories to marine teleost fish, for which data are currently limited. After explaining the new methods, with care to acknowledge their strengths and short fallings, the authors are able to assign a threat category to a huge proportion of fish for which IUCN currently are not able to assess. Moreover, the authors explore the consequences of their classifications, both in terms of where hotspots of extinction might be, and how they might change with this new kind of knowledge. They present the effectiveness of current protection based on knowledge before and after this method is applied and explore the how conservation planning might change with this new classification.

I was very impressed with this paper and feel that it makes an important, novel and interesting contribution to the field. The paper is presented clearly, has excellent visuals and would be of interest to a broad range of scientists, managers and policy makers.*

Thank you for these positive comments.

*R1#2 A few small things that I feel could use some work / clarification are as follows:

The discussion feels very long for the format of the journal and to me includes a lot of delving deeper into the results. I wonder if some of this detail could be pulled back into the results and then the discussion could more concisely consider the limitations, future directions and implications of the findings.*

We try our best to reduced the length of the discussion (by 4.5%) by removing a fullparagraph (line 329 to 343 in the previous version of the ms). Since referees 2 and 3 asked for a more in-depth discussion about our the caveats and the associated uncertainties of our approach, which we have developed in other parts of the discussion

*R1#3 In the methods, the fish occurrence data that drives the results comes from 2014 which is now 10 years old. I wonder if the authors could comment on the implications of this given that there could have been significant changes in species ranges or exploitation in the past 10 years?*

We acknowledge that fish occurrence data originate from 2014. However, updating the data set is a process that would take several months. We used the framework and data provided by Albouy et al. (2019) who developed a robust method to minimize errors in the original OBIS dataset, which involves the following steps:

- Extract data from OBIS,
- Clean the taxonomy.
- Clean the data by removing erroneous occurrences,
- Recreate range maps while controlling for depth,
- Merge with the GASPAR database (Parravicini et al. 2021),

We chose to work with this data set because it is the most accurate, even if not the most recent. OBIS is continuously aggregating new observations and it would thus be challenging to use these data to get range contractions for all species: species with no observations after 2014 in OBIS are not necessarily absent. For these reasons we believe that our data set is the most accurate one for all marine fish available today. We agree that there might have been some changes in species distribution over the last 10 years but we cannot overcome this limitation. We mention this point in the discussion lines (lines 321-326).

*Albouy, C. et al. The marine fish food web is globally connected. Nature Ecology Evolution 3, 1153-1161(2019).*

*Parravicini, V., Bender, M. G., Villéger, S., Leprieur, F., Pellissier, L., Donati, F. G. A., ... & Kulbicki, M. (2021). Coral reef fishes reveal strong divergence in the prevalence of traits along the global diversity gradient. Proceedings of the Royal Society B, 288(1961), 20211712.*

*R1#4 There is only a brief mention of climate change in the paper, which feels too little given the importance of climate as a driver for extinction in the coming decades. Could the authors expand on ways in which to better capture climate vulnerability into the assessment?*

We agree that climate vulnerability is an important aspect of species responses to global change and that it may modulate species extinction probabilities. Yet, Boyce et al. (2022) used a combination of species geographic ranges and traits to compute this climate vulnerability index which is related to the IUCN classification. Boyce et al. (2022) used the IUCN conservation status as one of the drivers of climate vulnerability. We believe that better capturing climate vulnerability into IUCN assessment would require species traits related to physiological responses to climate (i.e. metabolic rates, thermal optimum, reproductions, etc.) which are not available for most marine fishes. We added this perspective in our discussion lines 316-321.

*Boyce, D. G., Tittensor, D. P., Garilao, C., Henson, S., Kaschner, K., Kesner-Reyes, K., ... & Worm, B. (2022). A climate risk index for marine life. Nature Climate Change, 12(9), 854-862.*

*R1#5 I think this is a great paper and I look forward to see its publication*

Thank you for this positive feedback.

***Reviewer #2:*** *R2#1 This is an interesting, novel, timely, and important work. It adds to the growing literature on modelling threat status - albeit on thousands of species from a group that received to-date less evaluation attention - marine fishes. Nevertheless, I feel like some improvements could be made to the methods and their explanations.* 

Thank you for this positive feedback.

*R2#2 First, I would be interested in some more results and discussion of the 'erroneous' classification of your models. You modelled threat for thousands of species that have such classification yet did not explore patterns in these species. These modelled vs. 'real' categories could both provide interesting insights regarding your models' performance and potential gaps, and highlight species that do have IUCN non-NS status - but is potentially erroneous. I'd like to see Fig. 3 (or better yet a confusion matrix), of your classification of non-NS species. There could be some interesting patterns there. This is especially true if your two models' consensus is that a species should be classified differently than it currently is. Look at the 22-29% of 'error' classifications - first which direction they went, do they have particular attributes. The same goes for your 'modeled' NS species.*

Following this comment, we now provide in supplementary material plots of the proportion of false positive and false negative errors for both random forest and ANN models (See Figure S1). This analysis allows us to improve our results see lines 133-143.

Figure S1: Boxplot representing the percentages of True predictions (TP), False positives (FP) and False Negatives (FN) for each algorithm (RF and ANN). (RF) and the artificial neural network algorithm (ANN).

*R2#3* *Also, I suggest you calculate other classification performance statistics - F1, recall, precision. This will validate if your efforts to treat the class imbalance in your categories worked well. Ultimately if you predict all your non-NS species as non-threatened you'd get an accuracy of ~96%.*

As you suggested we also computed F1, recall and precision (that are of course linked to the relative proportion of false positives and false negatives relative to true positives). See figure S2 in supplementary material :

Figure S2: Boxplot representing performance statistics - Accuracy, F1, recall, precision scores of the random forest model (RF) and the artificial neural network algorithm (ANN).

*R2#4 Your mapping of the fishes distribution is simplistic. I understand that there is a major gap in knowledge regarding their distribution however If your grid your ranges to 1*1 degrees your minimum range for a species is 1*1 degree which can be an overestimate for many species. Also, it is unclear how this relates to you 50*50 km cells for the zonation analysis. Beyond this is seems like your grid contains non-equal area cells.*

All our analyses are at 10x10km resolution.

There was some confusion in our methods section so thank you for pointing this out. We used the 1x1 degree fish range maps and downscaled it at a 10x10km resolution to make all our spatial data (fish range maps & protected areas) at the same resolution. We modified our method section to make this clearer (see lines 441-447)

*R2#5 Have you conducted a sensitivity analysis on how your ranks for threatened species affects your zonation results (1 vs. 6)? Also have you considered ranking NS species differently to Non-threatened ones?  (say with the middle values between the other two categories)? There are accumulating evidence that DD and also NE species are more similar to threatened species in attributes and threats.*

We agree that it is important to rank the NS (now called DDNE) species differently than Non-threatened ones; which is what we did (DDNE were scored 2). We realize that we were not clear enough in our method and result sections on this point and have modified the two sections accordingly, lines 242-250 and lines 552-567. Thank you for highlighting this critical point.

The weights attributed to the different IUCN categories are subjective by definition (Lehtomäki & Moilanen, 2013). For example, performing a sensitivity analysis by using a null hypothesis (no weight difference between all IUCN categories) would simply make the zonation mirror the gradient of species richness which is basically not the aim of conservation strategies. We hardly see any simple alternative sensitivity analysis we could perform between these two extremes without adding even more suggestivity. Please also note that species were weighted proportionally to their IUCN Red List category following Pouzols et al. (2013) who assigned the following weights to least concern: 1, near threatened: 2, data deficient: 2, vulnerable: 4, endangered: 6, and critically endangered species: 8. Accordingly we fixed the weights to 1 for Non-Threatened, 2 for DDNE and 6 for Threatened species.

We better described the underlying hypothesis and objective of the zonation algorithm in the method section lines 244-246, 536-551.

Lehtomäki, J. & Moilanen, A. Methods and workflow for spatial conservation prioritization using Zonation. Environmental Modelling & Software 47, 128-137 (2013).

Pouzols, F.M., Toivonen, T., Di Minin, E., Kukkala, A.S., Kullberg, P., Kuusterä, J., Lehtomäki, J., Tenkanen, H., Verburg, P.H. & Moilanen, A. (2014) Global protected area expansion is compromised by projected land-use and parochialism. Nature, 516, 383–386

*R2#6 When imputing your trait values for species you, in essence, already incorporate phylogeny into your dataset indirectly. This should be acknowledged.*

We agree that species traits by essence incorporate phylogeny. We highlighted this important point in our discussion, see lines 352-353

*Minor comments
R2#7 Line 17 - you give an address for the "CEFE, Univ Montpellier, CNRS, EPHE-PSL University, IRD, Montpellier,*

*France" - but in the author list nobody is affiliated with it.*

We corrected it according to the reviewer’s comment.

*R2#8 Fig. 1 - The number of species in this figure is an underestimate for some groups. For example, according to 'the reptile database' (*[*http://www.reptile-database.org/db-info/SpeciesStat.html*](http://www.reptile-database.org/db-info/SpeciesStat.html)*) there are over 12,000 species of reptiles. This will bring the percentage of NS reptile species in your figure a lot. Amphibiaweb also has many more amphibian species, Birdlife for birds, and some other sources for mammals (see also Meiri et al. 2023).*

*Meiri S., Chapple D.G, Tolley K.A., Mitchell N., Laniado T., Cox N., Bowles P., Young B.E., Caetano G.H.O., Geschke J., Böhm M., & Roll U. 2023. Done but not dusted: reflections on the first global reptile assessment and priorities for the second. Biological Conservation, 278: 109879.*

Thanks for pointing that out. We updated the species number for birds, reptiles, mammals and amphibians using the reference that you provided and updated the figure 1 accordingly.

*R2#9 In general, I would suggest removing non-standard acronyms. Certainly 'NT' - which has a different meaning with respect to the IUCN Redlist - than used here.*

We removed all non standard acronyms from our text and figures. We now refer to Non-Threatened, Threatened species. We replace “NS” (Non Status) by “DDNE” (Data Deficient or Non Evaluated) to match with the IUCN Red List.

***Reviewer #3:*** *R3#0 This study builds a model that aims to predict IUCN status of marine fish by utilizing random forest and artificial neural network algorithms alongside occurrence data, traits, taxonomy, and human uses. It finds that the risk of extinction for marine fish is greater than estimated by IUCN. It then compares areas of hotspots for threatened-species compared to areas currently prioritized for conservation planning. These analyses are accompanied by neat, easily-understood figures. I find this a really interesting and useful study, utilizing RF and ANN algorithms to better understand threatened status where there is no IUCN status for understudied species.*

Thank you for these positive comments

*R3#1 However, I think that the low accuracy and method for combining the algorithms could be improved or at the very least accompanied with more caveats so that the reader understands the limitations of the predicted status. The low accuracy for both algorithms is slightly worrying and while I do understand that there were limitations with the number of threatened species available in the learning dataset, this does mean that the results should be presented with more caveats and uncertainties.*

We acknowledge that our accuracy is lower than other recent classifiers proposed for other taxa such as amphibians (85%, Borgelt et al. 2022) or orchids (84%, Zizka et al. (2021)). However, beyond accuracy itself, we added a layer of consensus for each algorithm (we ran 240 models and attributed a IUCN status only to species for which there was a consensus higher than 80%) which we believe increased the robustness of our predictions. We realized that our methods were not clear enough on this additional consensus step and modified our text accordingly, lines 297-298 491-512-. However, we agree that limitations and caveats associated with predictive accuracy need to be clearly stated and discussed, which we have strengthened in our discussion section lines 285-303, 491-512.

*“Our dataset was highly imbalanced; of the remaining 12,714 species, 324 were classified as Threatened, 7,750 as Non-Threatened, and 4,640 remained DDNE. Therefore, we divided the dataset into 24 down-sampled datasets with the 324 Threatened species and a different subset of 324 Non-Threatened species. First, we implemented Random Forest (RF). We ran RF with 10-fold cross-validation on each of the down-sampled datasets, resulting in a total of 240 RF models. The accuracy was on average 0.77 (See Fig S1 and S2). Then, we predict IUCN status for DDNE species for each of the 240 down-sampled datasets. We attributed a IUCN status only to species for which there was a consensus higher than 80% over the 240 RF models. For deep learning, the same framework, features and data sets as the RF approach have been used. We implemented an Artificial Neural Network (ANN) using cito (v1.1) R packages (Amesöder et al. 2024). The accuracy of the ANN was 0.70. We also ran 240 models for the 4,640 DDNE species and attributed a IUCN status only to species for which there was a consensus higher than 80% over the 240 ANN models.”*

*Amesöder, C., Hartig, F., & Pichler, M. (2024). ‘cito': an R package for training neural networks using ‘torch'. Ecography, e07143.*

*R3#2 Furthermore, I find the combination of machine and deep learning algorithms to be questionable - specifically with the 2nd step of the RF-ANN complementary decision tree (line 491: (ii) when one of the algorithm was not able to predict status but the second was able, the predicted status of this last one was assigned to the species). It makes sense that when both algorithms agree then that predicted status should be used, but if one algorithm simply doesn't report a status, I don't think that the assumption should be that the first algorithm is correct. It seems to me that the point of this step is a cross-check. If there is nothing else to cross-check with, shouldn't the species be assigned a No Status, as this is a form of divergence (as in option (iii))? Otherwise, this could lead to higher inaccuracy or muddy the overall dataset.*

Since we built a robust within-consensus framework (cross-check within each algorithm, see R3#1), we made the choice to present in the main text the complementary decision between algorithms allowing us to predict more species status than a between-consensus decision tree (1,249 more, 25% of the 4,640 DDNE). We also implemented the between-consensus decision tree (Figure S3) and found a qualitatively similar disproportionate increase in the number of threatened species which illustrates the robustness of our results. Overall, using two models rather than only one, as most predictive models presented in the literature do, is a strength of our approach: it leads to predicting less species status than by using only one model. Here we cannot provide a status for 573 species (12% of the 4,640 DDNE) for which predictions differed between the 2 models. Yet, we obtained more confidence in our predictions by combining results from RF and ANN models (see lines 296-303)

We agree however that this point should be clearer in both the methods, results and discussion. We modified our text accordingly (lines R3#2a, R3#2b and R3#2c). We also now propose to flag species with no consensus between models. We suggest we need more data for these species to improve the prediction as we already did for the remaining DDNE species (lines).

*R3#3 There is mention of a couple sensitivity tests that were conducted, but these results are not expanded on much. For example, there is mention of a sensitivity test conducted for the RF-ANN algorithm combination step (line 493) where both algorithms had to predict the same result and refers to Fig. 2. However, I believe Fig. 2 displays the method reported in the paper, not the sensitivity analysis version (which I believe is Fig S1?).  I think you could expand on this sensitivity test more to justify your chosen 3-option method as it seems quite a big assumption that if an algorithm doesn't predict a status, then just refer to the results of the second algorithm.*

See our response to R3#2. Thank you for noticing that we referred to the wrong figure line 493 in our previously submitted manuscript. We were referring to Fig. S1 indeed (now Fig. S3 in the revised manuscript).

*R3#4 There is also mention of a Zonation algorithm but the explanation is unclear and a bit too brief. Please expand on this - where do the 6 classification levels come from and how are they defined? There is again mention of a plotted sensitivity analysis (line 541) but I'm unsure which figure is associated with this - is it Fig. 7?*

In zonation algorithms, weights for species always need to be fixed (Lehtomäki & Moilanen, 2013), which we agree add some subjectivity to the analysis. A null hypothesis would be to treat all species equal and give all IUCN statuses the same weight. This would lead to the prioritization obtained through zonation to simply mirror species richness gradient. Our objective in using the zonation algorithm was to rank cells by conservation priority (given that IUCN Red List is used as an instrument for conservation planning) so we gave more weight to the Threatened species. Pouzols et al. (2014) assigned the following weights to least concern: 1, near threatened: 2, data deficient: 2, vulnerable: 4, endangered: 6, critically endangered: 8. Accordingly we fixed the weights to 1 for Non-Threatened, 2 for DDNE and 6 for Threatened species.

We made a better description of the zonation algorithm’s objective and underlying hypothesis in the method section lines 242-250 and lines 552-567.

We performed a sensitivity analysis giving more weight to species that have been evaluated by the IUCN than by our model. We weighted species as follows:

for species evaluated by IUCN, we kept a weight of 1 for Non-Threatened and 6 for Threatened species. Then, we fixed the weights of predicted species as a function of the average proportion of models (between RF and ANN) *p,* and obtained the final attributed status as follows : Threatened = 2 + *p* (rescaled between 2 and 5), Non-Threatened = 2 - *p* (rescaled between 1 and 2) and DDNE species = 2.

The results of this sensitivity analysis are presented in figure S7 of the revised manuscript.

Lehtomäki, J. & Moilanen, A. Methods and workflow for spatial conservation prioritization using Zonation. Environmental Modelling & Software 47, 128-137 (2013).

Pouzols, F.M., Toivonen, T., Di Minin, E., Kukkala, A.S., Kullberg, P., Kuusterä, J., Lehtomäki, J., Tenkanen, H., Verburg, P.H. & Moilanen, A. (2014) Global protected area expansion is compromised by projected land-use and parochialism. Nature, 516, 383–386

*R3#5 Overall I find this an important and interesting study that covers a gap in knowledge for threatened status of marine fishes and just needs a little more clarification on the methods and caveats for the low model accuracy.*

Thank you for your feedbacks
