# Supplementary material for: Inferring the extinction risk of marine fish to inform global conservation priorities
Source: PLoS Biol. 2024 Aug 29;22(8):e3002773. doi: 10.1371/journal.pbio.3002773 (PMC11361419; doi:10.1371/journal.pbio.3002773)
Supplement: Attachment — Submitted filename: Response.docx [file pbio.3002773.s004.docx]

Dear Dr. Roberts.

On behalf of my co-authors, I’m pleased to send you the revised version of our manuscript PBIOLOGY-D-23-03393R2, "*Inferring marine fish extinction risk to inform global conservation priorities*" now entitled “*Inferring the extinction risk of marine fish to inform global conservation priorities*”. We would like to thank you for your support and confidence throughout the revision process.

Please, see our response below.

Sincerely

Nicolas Loiseau

Thank you for your patience while we considered your revised manuscript "Inferring marine fish extinction risk to inform global conservation priorities" for publication as a Research Article at PLOS Biology. This revised version of your manuscript has been evaluated by the PLOS Biology editors and the Academic Editor.

Based on our Academic Editor's assessment of your revision, we are likely to accept this manuscript for publication, provided you satisfactorily address the following data and other policy-related requests.

IMPORTANT - please attend to the following:

a) Please change your Title slightly to: "Inferring the extinction risk of marine fish to inform global conservation priorities"

#We modified accordingly

b) There are some issues with English language/grammar throughout the manuscript, so you should re-read it carefully with this in mind; alternatively you may benefit from running it past a native English speaker, or enlist a professional editing service.

#A native English speaker has now re-read our manuscript (see in the Acknowledgment section). Correction are highlighted in the track-changes document.

c) Because the output of this study is likely to be of broad interest to people who do not have access to the appropriate computational tools, please provide a simple spreadsheet containing the inferred IUCN status of all ~4,600 species that you analysed, for non-R-conversant readers? (there may already be one in your Github deposition, but I couldn't find it). This should be uploaded as a supplementary Table, and cited in the manuscript.

#The table is now uploaded as a supplementary table and cited in the manuscript.

d) Many thanks for providing the underlying data and code in Github. Please could you confirm that it is sufficient to generate Figs 1, 3, 4, 5ABCD, 6AB, 7AB, and Figs S1-S8?

#I confirm that codes are sufficient to generate these figures.

e) Because Github depositions can be readily changed or deleted, please make a permanent DOI’d copy (e.g. in Zenodo) and provide this URL (see next point).
#We saved the Github deposition in Zenodo and provide the URL in the manuscript.

f) Please cite the location of the data clearly in all relevant main and supplementary Figure legends, e.g. “The data underlying this Figure can be found in <https://zenodo.org/records/XXXXXXXX>
#Done, see <https://zenodo.org/records/12783687>.

As you address these items, please take this last chance to review your reference list to ensure that it is complete and correct. If you have cited papers that have been retracted, please include the rationale for doing so in the manuscript text, or remove these references and replace them with relevant current references. Any changes to the reference list should be mentioned in the cover letter that accompanies your revised manuscript.
